# Distalization with Clear Aligners: Accuracy, Impact of Mini-Screws, and Clinical Outcomes

**DOI:** 10.3390/dj13070316

**Published:** 2025-07-14

**Authors:** Teresa Pinho, Diana Melo, Sofia Ferreira, Maria Gonçalves

**Affiliations:** 1 UNIPRO—Oral Pathology and Rehabilitation Research Unit, University Institute of Health Science (IUCS—CESPU), 4585-116 Gandra, Portugal; a28348@alunos.cespu.pt (D.M.); a28293@alunos.cespu.pt (S.F.); 2 UMIB—Multidisciplinary Biomedical Research Unit, Abel Salazar Institute of Biomedical Sciences (ICBAS), University of Porto, 4050-313 Porto, Portugal; 3 Associate Laboratory I4HB, Institute for Health and Bioeconomy, University Institute of Health Sciences (IUCS—CESPU), 4585-116 Gandra, Portugal; mprazeres.goncalves@iucs.cespu.pt; 4 UCIBIO—Applied Molecular Biosciences Unit, Translational Toxicology Research Laboratory, University Institute of Health Sciences (1H-TOXRUN, IUCS—CESPU), 4585-116 Gandra, Portugal

**Keywords:** distalization, Invisalign, clear aligners, mini-screws, PAR index

## Abstract

**Background:** Distalization is a fundamental orthodontic strategy for correcting Class II and Class III malocclusions, particularly in cases where specific dental or skeletal conditions favor its application. Recent technological advances have enabled complex dental movements to be performed using clear aligners, aided by digital planning platforms such as ClinCheck^®^. **Methods:** This retrospective study aimed to evaluate the accuracy of ClinCheck^®^ in predicting molar and canine distalization outcomes with the Invisalign^®^ system and to identify clinical factors influencing treatment predictability. Thirty patients with complete permanent dentition and at least 2 mm of programmed distalization were selected. Planned movements were extracted from the Invisalign^®^ Doctor Site and compared to achieved outcomes using Geomagic^®^ Control X™ software. Occlusal improvements were assessed using the *Peer Assessment Rating (PAR) index*. **Results:** The results revealed significant discrepancies between the programmed and achieved distalization, with mean deviations greater than 1 mm in both arches. Skeletal anchorage with *mini-screws* significantly improved distalization outcomes in the maxillary arch; however, no significant effect was observed in the mandibular arch. Additionally, no significant associations were found between distalization outcomes and skeletal pattern (*ANB angle*) or *facial biotype*. **Conclusions:** Clear aligners are effective in achieving substantial occlusal improvements, particularly when combined with personalized digital planning and supplementary strategies such as skeletal anchorage. Mandibular cases demonstrated greater reductions in PAR scores, emphasizing the potential of aligners in complex distalization treatments.

## 1. Introduction

Distalization is a key orthodontic strategy for correcting Class II and Class III malocclusions and can serve as a camouflage approach in cases involving mild to moderate skeletal discrepancies [1,2]. This movement involves the posterior displacement of teeth to create anterior space, often avoiding the need for extractions [3]. Maxillary molar distalization is particularly effective for Class II correction, while mandibular distalization, although less frequently indicated, can contribute to the management of mild Class III cases [1,4]. While methods such as mandibular advancement are also employed in the correction of Class II malocclusions, as evidenced by the literature [5,6], distalization is particularly indicated in cases where specific dental or skeletal conditions favor its application.

The growing demand for discreet and comfortable orthodontic solutions has contributed to the widespread adoption of clear aligners [7,8]. Initially developed for minor tooth movements, systems like Invisalign^®^ have evolved through technological innovations, such as attachments, precision cuts, and CAD-CAM digital planning tools like ClinCheck^®^ to enable the execution of more complex movements, including distalization [9,10].

Advancements in 3D scanning technologies and analysis software, such as Geomagic^®^, in addition to cephalometric evaluation techniques, have enabled more precise assessments of treatment predictability. Three-dimensional superimpositions enable detailed comparisons to be made between programmed and achieved movements, and cephalometry provides valuable insight into skeletal and dental change [11].

Recent studies demonstrate that Invisalign^®^ can achieve maxillary molar distalization of approximately 2.0 mm, typically without significant vertical alterations, and often with discrepancies from planned movements of less than 0.5 mm [12,13]. Furthermore, the use of skeletal anchorage devices, such as *mini-screws*, has been shown to enhance the predictability and stability of distal movements, especially in complex or asymmetric clinical scenarios [1,14].

Despite technological progress, individual anatomical characteristics, including arch length discrepancies, initial crowding, and *skeletal pattern*, continue to influence the effectiveness of distalization [3]. This underscores the necessity of personalized treatment planning. The *Peer Assessment Rating (PAR)* index is a valuable tool for quantifying initial malocclusion complexity, although its predictive value regarding treatment outcomes remains to be fully clarified [15,16].

Considering the therapeutic potential of distalization in managing both Class II and Class III malocclusions, evaluating the predictability of digital planning tools is critical.

This retrospective study aimed to evaluate the accuracy and clinical outcomes of molar distalization with clear aligners and the impact of *mini-screws*, *facial biotype*, and *skeletal pattern* on treatment results. Planned and achieved tooth movements were assessed by comparing digital models and clinical records before and after treatment.

## 2. Materials and Methods

### 2.1. Study Design

This is a quantitative, comparative, and observational retrospective cohort study. It was conducted per the Declaration of Helsinki (1975, revised 2013) and was approved by the Ethics Committee of the University Institute of Health Sciences (IUCS-CESPU) under protocol number 03/CE-IUCS/2025. The study also adhered to the CONSORT guidelines (http://www.consort-statement.org; accessed on 16 December 2019). All participants provided informed consent after receiving a thorough explanation and evaluation of the study.

### 2.2. Samples and Eligibility Criteria

Thirty patients with complete permanent dentition (up to the second molars, and third molars, if not already absent, were previously extracted in cases where distalization was planned, either in the maxillary or mandibular arch) undergoing orthodontic treatment with clear aligners were selected from a private clinic in Northern Portugal. All patients were treated by the same orthodontist, a Diamond Invisalign Provider (T.P.).

Inclusion criteria required a minimum of 2 mm of posterior distalization to focus on moderate and complex cases. Patients were instructed to wear the aligners for 20–22 h per day, removing them only for oral hygiene and meals, and to change them weekly. Compliance was verbally confirmed at each appointment. Detailed eligibility criteria are summarized in Table 1.

### 2.3. Data Collection Procedures

#### 2.3.1. Digital Acquisition

Digital intraoral scans were taken before and after treatment using the iTero^®^ Element 5D Plus scanner (Align Technology, Tempe, AZ, USA). Planned tooth movements were retrieved from the Invisalign^®^ Doctor Site using ClinCheck^®^ Pro 6.0 software.

#### 2.3.2. Measurement of Distalization

**Figure 1:**
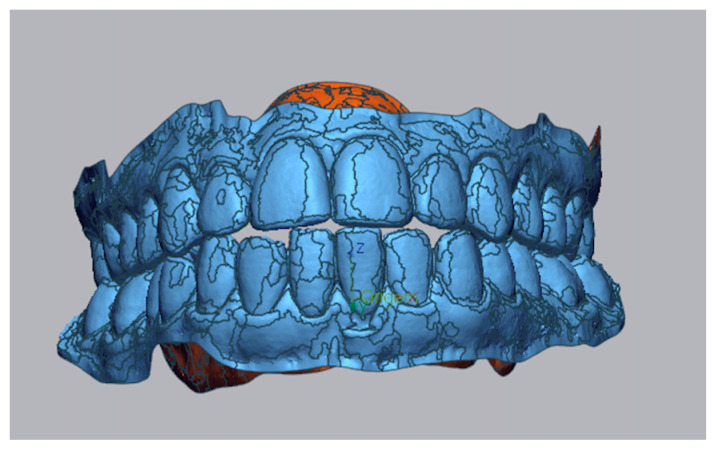
In blue: Pre-treatment STL model with generated 3D mesh.

The extent of distalization was evaluated following a standardized protocol using Geomagic^®^ Control X™ software 2023.1.0 (Oqton, Ghent, Belgium):

Step I: Importing the pre-treatment model and generation of a 3D mesh, Figure 1.

**Figure 2:**
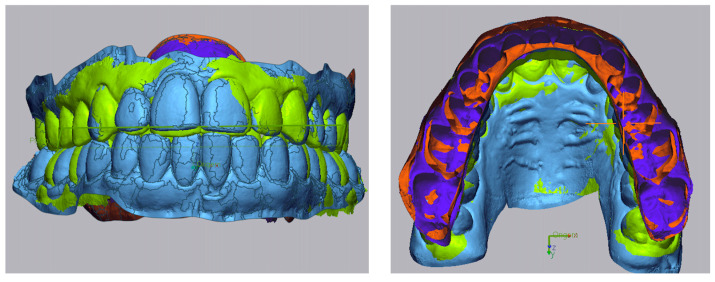
Superimposition of pre– (in blue) and post–treatment (in green) models based on palatal rugae.

Step II: Figure 2 shows the superimposition of the post-treatment model on the pre-treatment model using “Initial Alignment” and “Best Fit Alignment,” based on palatal rugae as stable reference structures [17].

**Figure 3:**
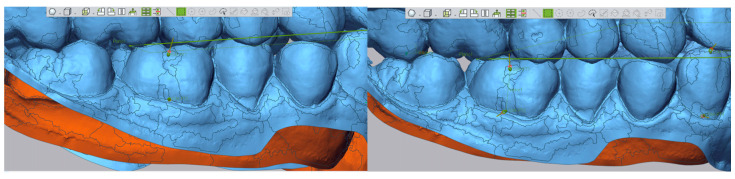
Tracing of molar and canine long axes.

Step III: Construction of long axis vectors for the first molars and canines, based on cusp and gingival points [18], Figure 3.

**Figure 4:**
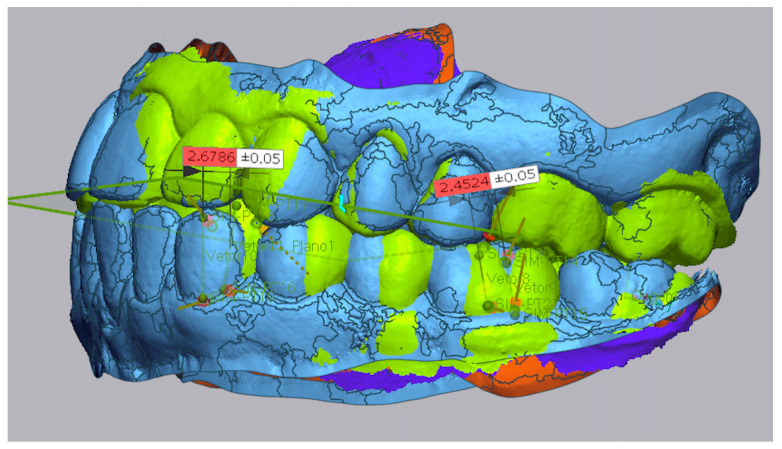
Measurements of molar and canine distalization.

Step IV: Figure 4 shows the measurement of linear displacements between pre– (in blue) and post–treatment (in green) vectors to quantify distalization.

#### 2.3.3. Clinical and Cephalometric Data

Patient demographics (age and sex) and cephalometric parameters (overjet, overbite, facial biotype, and ANB angle) were collected from clinical records.

#### 2.3.4. Occlusal Assessment

The *Peer Assessment Rating (PAR)* index was used to quantify the severity of initial malocclusion and post-treatment improvements. Weighted *PAR* scores were calculated, and the percentage of occlusal correction was determined using the formula [15]:

$$\%PAR=\frac{PAR\;T1-PAR\;T2}{PAR\;T1}\times 100\%$$

Patients were classified according to the degree of improvement: no improvement/worsening (<30%), moderate improvement (30–70%), and significant improvement (≥70%) [15].

Based on the degree of improvement, cases are categorized into three groups: no improvement or worsening (<30% improvement), moderate improvement (30–70%), and significant improvement (≥70%) [15].

To collect data on the planned distalization, the tooth movements were extracted from the Invisalign^®^ Doctor Site based on the first set of aligners.

### 2.4. Statistical Analysis

Statistical analyses were performed using IBM^®^ SPSS^®^ Statistics for Windows, version 29.0 (IBM Corp., Armonk, NY, USA). Descriptive statistics, including absolute and relative frequencies, means, standard deviations, medians, and minimum and maximum values, were calculated for all variables. The assumption of normality was evaluated using the Shapiro–Wilk test. As normality was confirmed, parametric tests were employed for subsequent analyses. Comparisons of categorical demographic and clinical variables between groups were performed using the chi-square test or Fisher’s exact test when appropriate. Differences between programmed and achieved distalization movements, and changes in the *Peer Assessment Rating (PAR)* index from baseline to post-treatment, were assessed using paired-samples t-tests. Independent-samples t-tests were conducted to compare distalization outcomes between individuals treated with and without skeletal anchorage (*mini-screws*) and evaluate the difference in *PAR* index reductions between the maxillary and mandibular distalization groups. To assess the influence of *facial biotype* on distalization outcomes, a one-way analysis of variance (ANOVA) was applied, followed by Bonferroni post hoc tests for multiple comparisons. Effect sizes for ANOVA were estimated using eta squared (η^2^), categorized as small (η^2^ = 0.01), medium (η^2^ = 0.06), and large (η^2^ = 0.14). Finally, the association between programmed distalization and skeletal pattern (*ANB angle*) was assessed using Pearson’s correlation coefficient. The significance level for all statistical tests was set at *p* < 0.05.

## 3. Results

### 3.1. Characteristics of the Clinical Study Sample

A total of 30 patients were included, with 16 cases involving maxillary distalization and 14 involving mandibular distalization. The mean age was 20.94 ± 2.9 years for the maxillary group and 28.79 ± 9.92 years for the mandibular group.

Demographic and clinical characteristics, including sex distribution, facial biotype, skeletal class, and mini-screws usage, are summarized in Table 2.

### 3.2. Planned Treatment Series and Number of Aligners

The mean number of aligner series was higher in the maxillary group (3.69 ± 1.14) compared to the mandibular group (2.86 ± 1.03). The average number of aligners planned and used is detailed in Table 3.

### 3.3. Maxillary and Mandibular Distalization Movements

Planned distalization movements were greater in the maxillary arch compared to the mandibular arch for both canines (4.11 ± 0.99 mm vs. 3.12 ± 1.03 mm) and molars (4.35 ± 1.23 mm vs. 3.89 ± 1.30 mm). However, in both arches, the achieved movements were lower than planned, with mean values of 2.62 ± 1.28 mm (canines) and 2.73 ± 1.54 mm (molars) in the maxillary arch, and 2.07 ± 1.02 mm (canines) and 2.51 ± 1.21 mm (molars) in the mandibular arch (Table 4).

### 3.4. Comparison of Planned Maxillary and Mandibular Distalization with That Obtained at the End of Treatment

Planned distalization movements were greater in the maxillary arch compared to the mandibular arch for both canines (4.11 ± 0.99 mm vs. 3.12 ± 1.03 mm) and molars (4.35 ± 1.23 mm vs. 3.89 ± 1.30 mm). However, the achieved movements were significantly lower than planned in both arches, with values of 2.62 ± 1.28 mm (canines) and 2.73 ± 1.54 mm (molars) in the maxillary arch, and 2.07 ± 1.02 mm (canines) and 2.51 ± 1.21 mm (molars) in the mandibular arch (Table 5). Paired *t*-tests confirmed significant differences between planned and achieved movements (*p* < 0.05), with mean discrepancies ranging from 1.05 mm to 1.62 mm for all teeth analyzed (Table 5).

### 3.5. Correlation with Skeletal Pattern

Programmed distalization movements of the canines at the maxillary level are strongly, statistically significantly, and positively related to the distalization movements of the molars (*r* = 0.832; *p* < 0.01). The ANB angle exhibits a weak positive correlation with canine distalization movements (*r* = 0.200) and a very weak negative correlation with molar distalization (*r* = −0.070), though neither reaches statistical significance. At the mandibular level, the programmed distalization movements of the canines are moderately and positively related to the distalization movements of the molars (*r* = 0.497), though this is not statistically significant. The ANB angle shows a weak positive correlation with canine and molar movements (*r* = 0.073 and *r* = 0.007, respectively), but this is not statistically significant (Table 6).

At the maxillary level, the programmed distalization movements of the canines are strongly and significantly positively correlated with the distalization of the molars (*r* = 0.832; *p* < 0.01). The ANB angle showed a weak positive correlation with canine movement (*r* = 0.200) and a negligible negative correlation with molar movement (*r* = −0.070), neither of which was statistically significant. At the mandibular level, canine distalization was moderately positively correlated with molar distalization (*r* = 0.497), though this did not reach statistical significance. The ANB angle demonstrated very weak positive correlations with both canine (*r* = 0.073) and molar (*r* = 0.007) movements, also without statistical significance.

### 3.6. Comparison of Planned and Achieved Maxillary and Mandibular Distalization Between Individuals with and Without Mini-Screws (Independent Samples T-Test)

Table 7, in the maxillary arch, patients with mini-screws showed higher mean planned distalization values for canines (4.72 ± 0.86 mm vs. 3.83 ± 0.96 mm) without statistical significance, while for molars, the planned distalization was significantly greater with mini-screws (5.32 ± 1.04 mm vs. 3.92 ± 1.08 mm; *p* = 0.029). The achieved distalization was also significantly greater in individuals with mini-screws for both canines (*p* = 0.006) and molars (*p* = 0.031). In the mandibular arch, although the mean values were higher in patients with mini-screws for both the planned and achieved distalization, the differences were not statistically significant.

### 3.7. Comparison of Planned Maxillary and Mandibular Distalization Obtained at the End of Treatment According to Facial Biotype

A subsequent comparison of the distalization movements exhibited by subjects across various facial biotypes revealed no statistically significant disparities, neither in the programmed nor in the achieved distalization movements at the maxillary level. However, the hyperdivergent group exhibited higher mean values of programmed distalization for both canines (4.72 ± 1.56 mm) and molars (4.66 ± 1.28 mm). Regarding the achieved distalization, the mean values were comparable across groups, with hypodivergent individuals exhibiting slightly higher canine distalization (2.76 ± 1.70 mm) and hyperdivergent individuals demonstrating the highest molar distalization (2.86 ± 1.41 mm).

In a similar vein, the mandibular arch exhibited no statistically significant differences between biotypes. The hypodivergent group demonstrated the highest programmed distalization values for both canines (4.90 mm) and molars (5.20 mm), although it is worth noting that this subgroup included only one patient. Among the remaining biotypes, normodivergent individuals exhibited higher mean programmed distalization values compared to hyperdivergent individuals. For the achieved movements, higher mean values were observed in the hyperdivergent group for both canines (2.50 ± 1.24 mm) and molars (2.94 ± 1.57 mm).

### 3.8. PAR Index at the Maxillary and Mandibular Levels Before and After Treatment

Table 8 presents the mean values of the PAR (Peer Assessment Rating) index at T0 (start of treatment) and TFinal (end of treatment) for both the maxillary and mandibular arches. In the maxillary arch, a statistically significant reduction in the PAR index was observed, decreasing from 20.81 ± 6.88 at T0 to 3.06 ± 3.60 at TFinal, with a mean difference of 17.75 points (*p* < 0.001). Similarly, in the mandibular arch, the PAR index dropped from 19.30 ± 7.80 to 0.85 ± 1.70, corresponding to a mean difference of 18.43 points (*p* < 0.001).

Table 9 compares the PAR index and the percentage of reduction achieved between the maxillary and mandibular arches. Before treatment, the mean PAR values were similar for both arches, 20.81 ± 6.88 for the maxilla and 19.30 ± 7.80 for the mandible. After treatment, although both arches showed a significant decrease in the PAR index, the reduction was significantly greater in the mandibular arch (0.85 ± 1.70) compared to the maxillary arch (3.06 ± 3.60) (*p* = 0.046). Regarding the percentage reduction in the PAR index, the mandibular arch showed a significantly greater improvement (97.16 ± 5.73%) than the maxillary arch (86.76 ± 15.07%) (*p* = 0.026).

## 4. Discussion

Clear aligners represent a discreet and effective orthodontic modality for dental alignment, offering aesthetic advantages without compromising clinical outcomes [7,10]. However, their predictability in achieving complex movements such as molar and canine distalization remains under scrutiny [3].

In this study, clinically relevant distalization was achieved in both arches, yet deviations between programmed and achieved movements consistently exceeded 1 mm. In the maxillary arch, mean deviations were 1.49 ± 1.28 mm for canines and 1.62 ± 1.36 mm for molars. In the mandibular arch, deviations were 1.05 ± 1.31 mm for canines and 1.38 ± 1.29 mm for molars. These results highlight the limited predictability of ClinCheck^®^ for distalization. Our results are in line with the studies conducted by Taffarel et al. and Li et al. [19,20].

The decision to use mini-screws was not based solely on the amount of distalization, but rather on biomechanical complexity and the absence of reciprocal anchorage [1]. This was particularly relevant in Class III cases, where distalization was combined with retroclined lower incisors, resulting in all movements directed posteriorly, and in Class II cases with pronounced upper incisor proclination, where simultaneous anterior retraction and posterior distalization were required. In both situations, the lack of opposing forces increased unpredictability in movement, justifying the use of skeletal anchorage.

The results of this study indicate that skeletal anchorage with mini-screws significantly enhanced the amount of achieved distalization in the maxillary arch, particularly in both canines (*p* = 0.006) and molars (*p* = 0.031). The mean differences were clinically relevant (1.75 mm for canines and 1.74 mm for molars), and the confidence intervals did not include zero, reinforcing the significance of these findings. Planned distalization was also greater in the maxillary group with mini-screws, especially for molars (*p* = 0.029), suggesting that clinicians felt more confident in programming larger movements when skeletal anchorage was planned.

In contrast, mandibular distalization did not show statistically significant improvement with mini-screws, likely due to anatomical constraints (e.g., dense bone and limited space) [21] and the fact that all cases involved Class III skeletal patterns. These patterns inherently limit the biological feasibility of extensive distalization.

Nonetheless, despite the lack of statistical significance, a clinically satisfactory outcome was achieved in the end, with the establishment of a Class I occlusion as assessed by the PAR index, indicating effective compensation for the underlying skeletal discrepancy.

Furthermore, when a substantial amount of distalization is required to correct sagittal discrepancies in Class III patients, orthognathic surgery should be considered instead of excessive orthodontic compensation. Attempting to manage these cases with distalization alone may exceed the biological limits of movement and compromise long-term stability [1].

Notably, neither skeletal pattern, as measured by the ANB angle, nor facial biotype showed significant effects on distalization outcomes. This suggests that, when supported by individualized planning and biomechanical optimization, clear aligner therapy can deliver consistent and effective results across different craniofacial patterns.

The effectiveness of the treatment was objectively confirmed through the Peer Assessment Rating (PAR) index, with a substantial reduction in malocclusion severity observed [16]. The maxillary distalization group achieved a mean PAR depletion of 86.76%, while the mandibular group achieved 97.16%; this difference was statistically significant (*p* = 0.026). These results demonstrate that, despite the mechanical limitations of replicating the planned distalization, the clinical outcomes were highly successful in terms of both function and aesthetics.

It is important to note that the overall complexity of the case was also evaluated using the PAR index, which provides an objective assessment of occlusal irregularity. However, the indication for mini-screws was not guided by the PAR score, but rather by biomechanical considerations, specifically the degree of movement unpredictability and the absence of reciprocal anchorage. Therefore, the clinical complexity that justified skeletal anchorage was distinct from the occlusal complexity measured by the PAR.

Overall, the findings emphasize the importance of having realistic clinical expectations when planning distalization with aligners. To optimize outcomes, adjunctive strategies such as skeletal anchorage, close monitoring of treatment, and individualized staging are essential. Future prospective studies incorporating objective compliance monitoring and larger sample sizes are necessary to refine prediction models and optimize biomechanics for complex movements.

Although the sample size of 30 individuals was adequate for a preliminary analysis, it is relatively small and may limit the generalization of the results. Moreover, as all participants were recruited based on clinic visits, the sample should be considered a convenience sample. In this context of specific clinical issues, an a priori power analysis was not feasible and therefore was not conducted. Future studies with larger, more diverse samples and greater control of clinical variables are needed to confirm and expand these results.

## 5. Conclusions

Clear aligners can be used to distalize the molars and canines in moderate to complex Class II and Class III cases. However, the predictability of these movements is limited, as planned and achieved outcomes often differ by more than 1 mm.

Nevertheless, substantial clinical improvement was demonstrated through significant reductions in the PAR index, particularly in the mandibular group (97.16%), despite the lack of statistically significant skeletal anchorage effect in the lower arch.

Skeletal anchorage significantly improved the amount and predictability of distalization in the maxillary arch, especially for canines and molars, supporting its use in complex biomechanical cases with absent reciprocal anchorage.

Skeletal pattern and facial biotype had no significant influence on treatment outcomes, reinforcing the relevance of individualized digital planning and biomechanical control.

Adjunctive strategies, such as skeletal anchorage, customized staging, and realistic goals, remain essential to optimize distalization with aligners, especially in borderline cases.

## Figures and Tables

**Table 1:** Inclusion and exclusion criteria for the study.

Inclusion Criteria	Exclusion Criteria
- Full permanent dentition (up to the second molar) who underwent orthodontic treatment with Invisalign^®^. - Available and complete cephalometric analysis. - Programmed distalization movement of at least 2 mm, classified as moderate, in at least one of the hemi-arches, presented in the Invisalign^®^ Doctor Site. - Completed orthodontic treatment.	- Missing teeth, unerupted teeth, or congenital absence of permanent teeth up to the second molar. - Individuals with cognitive or neurological disorders, with identified syndromes, history of trauma and/or tumors in the head and neck, and metabolic diseases that affect the joints and/or muscles. - Individuals who were being treated with anti-inflammatories, analgesics, or psychiatric medication.

**Table 2:** Demographic and clinical characteristics of the 2 groups.

		Group Assessed at Maxillary Level (n = 16)	Group Assessed at Mandibular Level (n = 14)
		N (%)	N (%)
	Age (Mean ± SD)	20.4 ± 1.9	28.79 ± 9.92
Sex	Feminine	12 (57.1)	9 (42.9)
	Masculine	4 (44.4)	5 (55.6)
Facial biotype	Hypodivergent	7 (87.5)	1 (12.5)
	Hyperdivergent	5 (41.7)	7 (58.3)
	Normodivergent	4 (40.0)	6 (60.0)
Class	Class I	3 (33.3)	6 (66.7)
	Class II	13 (100.0)	0 (0.0)
	Class III	0 (0.0)	8 (100.0)
Overjet	Decreased	1 (100.0)	0 (0.0)
	Increased	10 (100.0)	0 (0.0)
	Normal	5 (26.3)	14 (73.7)
Overbite	Decreased	0 (0.0)	7 (100.0
	Normal	5 (100.0)	0 (0.0)
	Increased	11 (61.1)	7 (38.9)
Mini-screws	No	11 (64.7)	6 (35.3)
	Yes	5 (38.5)	8 (61.5)
Skeletal class	Class I	9 (64.3)	5 (35.7)
	Class II	6 (100.0)	0 (0.0)
	Class III	1 (10.0)	9 (90.0.0)

Note: Data are summarized as Mean ± SD, frequency, and percentages.

**Table 3:** Descriptive statistics for the number of treatment series and maxillary and mandibular aligners.

		Mean ± s.d	Minimum	Maximum
Maxillary	Number of treatment series	3.69 ± 1.14	2	5
	Number of aligners first series	52.19 ± 9.80	36	79
Mandibular	Number of treatment series	2.86 ± 1.03	1	4
	Number of aligners first series	57.14 ± 15.61	37	99

Note: Data are summarized as Mean ± SD, minimum, and maximum.

**Table 4:** Descriptive measures of distalization movements.

		Mean ± SD	Median	Minimum	Maximum
Maxillary					
Planned distalization	Canines	4.11 ± 0.99	4.10	2.70	5.90
	Molars	4.35 ± 1.23	4.10	2.80	6.20
Achieved at the end of treatment	Canines	2.62 ± 1.28	2.46	0.82	6.03
	Molars	2.73 ± 1.54	2.46	1.27	7.24
Mandibular					
Planned distalization	Canines	3.12 ± 1.03	2.95	1.50	4.90
	Molars	3.89 ± 1.30	3.65	2.10	6.20
Achieved at the end of treatment	Canines	2.07 ± 1.02	1.77	1.20	4.72
	Molars	2.51 ± 1.21	2.08	0.92	4.86

Note: Data are summarized as Mean ± SD, median, minimum, and maximum.

**Table 5:** Comparison of planned and achieved maxillary and mandibular distalization movements at the end of treatment.

			Mean ± SD	T0–Tfinal (mm)	CI 95%	*p*-Value
Maxillary	Canines	T0	4.11 ± 0.99	1.49 ± 1.28	[0.82–2.16]	<0.001
		Tf	2.62 ± 1.28			
	Molars	T0	4.36 ± 1.23	1.62 ± 1.36	[0.90–2.35]	<0.001
		Tf	2.73 ± 1.54			
Mandibular	Canines	T0	3.12 ± 1.03	1.05 ± 1.31	[0.9–1.81]	0.010
		Tf	2.07 ± 1.02			
	Molars	T0	3.89 ± 1.30	1.38 ± 1.29	[0.64–2.13]	0.001
		Tf	2.51 ± 1.21			

Note: Data are summarized as Mean ± SD;, mean difference, 95% CI, and *p*-value, derived from the paired *t*-test.

**Table 6:** Relationship between planned distalization movements and ANB angle.

Maxillary Planned Distalization	Canines	Molars
Molars	0.832 **	-
ANB angle	0.200	−0.07
**Mandibular Planned Distalization**		
Molars	0.497	
ANB angle	0.073	0.007

Note: Data are summarized as the Pearson’s correlation coefficient and *p*-value. ** *p* < 0.001

**Table 7:** Comparison of planned and achieved maxillary and mandibular distalization between individuals with and without mini-screws.

		Mini-Screws				
		No	Yes			
		Mean ± SD	Mean ± SD	Mean Difference	CI 95%	*p*-Value
MAXILAR						
Planned Movements	Canines	3.83 ± 0.96	4.72 ± 0.86	−0.88 ± 0.50	[−1.96; −0.19]	0.099
	Molars	3.92 ± 1.08	5.32 ± 1.04	−1.40 ± 0.58	[−2.64; −0.16]	0.029
Achieved Movements	Canines	2.08 ± 0.81	3.82 ± 1.37	−1.75 ± 0.54	[−2.91; −0.58]	0.006
	Molars	2.19 ± 0.90	3.92 ± 2.07	−1.74 ± 0.72	[−3.28; −0.18]	0.031
MANDIBULAR						
Planned Movements	Canines	2.57 ± 0.63	3.54 ± 1.10	−0.97 ± 0.50	[−2.07; 0.13]	0.078
	Molars	3.15 ± 0.84	4.45 ± 1.34	−1.30 ± 0.63	[−2.67; 0.07]	0.061
Achieved Movements	Canines	1.88 ± 0.67	2.22 ± 1.25	−0.34 ± 0.57	[−1.57; 0.90]	0.562
	Molars	2.19 ± 1.30	2.75 ± 1.18	−0.56 ± 0.66	[−2.01; 0.88]	0.413

Note: Data are summarized as the Mean ± SD, mean difference, 95% CI, and *p*-value, derived from the independent *t*-test.

**Table 8:** Changes in PAR index at the maxillary and mandibular levels.

	T0 (Mean ± SD)	TFinal (Mean ± SD)	Diff (T0–TFinal)	*p*-Value
Maxillary				
PAR index	20.81 ± 6.88	3.06 ± 3.60	17.75	<0.001
Mandibular				
PAR index	19.30 ± 7.80	0.85 ± 1.70	18.43	<0.001

Note: Data are summarized as the Mean ± SD, mean difference, and *p*-value, derived from the paired *t*-test.

**Table 9:** Comparison of PAR index and percentage reduction between maxillary and mandibular arches.

		Mean ± SD	Mean Diff.	*p*-Value
PAR index before	Maxillary	20.81 ± 6.88	1.53	0.573
	Mandibular	19.30 ± 7.80		
PAR index after	Maxillary	3.06 ± 3.60	2.21	0.046
	Mandibular (n = 14)	0.85 ± 1.70		
Percentage reduction	Maxillary (n =16)	86.76 ± 15.07	10.41	0.026
	Mandibular (n = 14)	97.16 ± 5.73		

Note: Data are summarized as the Mean ± SD, mean difference, 95% CI, and *p*-value, derived from the paired *t*-test.

## Data Availability

The data presented in this study are available on request from the corresponding author.
